# Efficacy and Safety of Intravenous Insulin in Treatment of Patient With Diabetic Ketoacidosis: A Systematic Review and Meta-Analysis

**DOI:** 10.7759/cureus.30721

**Published:** 2022-10-26

**Authors:** Kareema S Alshurtan, Osama Alnizari, Hadi Aldarwish, Ali A Al-Tufaif

**Affiliations:** 1 Internal Medicine, University of Hail College of Medicine, Hail, SAU; 2 Medicine, University of Hail College of Medicine, Hail, SAU

**Keywords:** subcutaneous insulin, intravenous insulin, diabetic coma, diabetic ketoacidosis, diabetes mellitus

## Abstract

The most common acute hyperglycemic emergency is diabetic ketoacidosis (DKA). DKA is one of the leading causes of Type 1 diabetes (T1D) related deaths in people aged 30 and under. In this meta-analysis, the Overall use of IV insulin in patients with mild/moderate vs. severe diabetic ketoacidosis was compared in randomized controlled trial articles from January 2011 to December 2021 using EMBASE, Medline, and CENTRAL. Only 8 of 3258 studies met the inclusion criteria. This review shows that intravenous insulin can significantly decrease plasma glucose and potassium levels in mild/moderate cases and severe cases. However, it can decrease the resolution time of acidosis more quickly in mild/moderate cases than in severe cases. In the current meta-analysis, the use of IV insulin is secure and efficient. There was no discernible difference in the effectiveness of IV insulin between mild/moderate and severe DKA.

## Introduction and background

Diabetes type 1 (T1D) is an autoimmune disease characterized by immune-mediated pancreatic beta-cell destruction, resulting in the limitation of the abnormal production and secretion of insulin [1]. T1D constitutes 5%-10% of all diabetes cases, with a global prevalence of 9.5% (15 per 100,000 people) [2]. The most common acute hyperglycemic emergency in diabetic patients is diabetic ketoacidosis (DKA). [3]. A systematic review reported that the incidence of DKA varies from 0 to 128 per 1000 person-year. DKA is more pronounced in young patients, women, and non-white individuals [4]. DKA symptoms include polyuria, polydipsia, vomiting, weight loss, stomach pain, and exhaustion. Uncontrolled diabetes can cause DKA [5]. Literature suggests that 54%-76% of all T1D-related deaths under 30 years of age are attributed to DKA [6].

The management of DKA includes the infusion of 1 liter of 0.9% sodium chloride over one hour, ensuring a potassium level above 3.3 mEq/L, and initiating insulin therapy [7]. To treat kids with diabetic ketoacidosis, a continuous intravenous insulin infusion at the recommended dose of 0.1 units/kg/h is advised [8]. The insulin injection increases peripheral tissues' ability to use glucose, reduces gluconeogenesis and glycogenolysis, and inhibits ketogenesis [9].

Compared to continuous intravenous insulin, the literature suggests that subcutaneous insulin infusion offers a feasible alternative for mild DKA. To treat mild to moderate DKA in adults, Andrade-Castellans et al. compare subcutaneous rapid-acting insulin analogues to conventional intravenous insulin. Their findings were not strong enough to predict the effectiveness of subcutaneous insulin [10]. Several meta-analyses and systematic reviews were published on this subject; however, the results were not consistent [11,12]. The literature suggests that intravenous insulin infusion is a superior method to treat DKA than subcutaneous insulin infusion; despite that, patients on intravenous insulin should be admitted to the intensive care unit for close mongering [13]. We were unable to find any previously published meta-analysis that assesses the safety and efficacy of intravenous insulin in treating patients with diabetic ketoacidosis.; therefore, our current meta-analysis purpose was to evaluate the efficacy and safety of intravenous insulin in the treatment of patients with diabetic ketoacidosis.

## Review

Method

Data Sources and Search Strategy

The Preferred Reporting Items for Systematic Review and Meta-Analyses (PRISMA) criteria were followed for this systematic review and meta-analysis [14]. An electronic search from PubMed/Medline, Cochrane Trial register, and Google scholar was conducted from January 2011 to 11 December 2021, using the search string: (diabetes OR DM OR T1D) AND (diabetic ketoacidosis OR DKA OR ketoacidosis) AND (intravenous insulin OR IV insulin OR insulin) AND (safety) AND (efficacy). In addition, we manually screened the cited articles of previous meta-analyses, cohort studies, and review articles to identify any relevant studies.

Study Selection

All studies were included if they met the following eligibility criteria, which can be described as PICOS: 1) P (Patients): Diabetes Ketoacidosis patients (DKA); 2) I (Intervention): Intravenous insulin; 3) C (Control): none; 4) O (Outcome): effect of Intravenous Insulin in DKA patients; 5) S (Studies): Cohorts and Randomized Controlled Trials published in English.

Literature Search Results

**Figure 1:**
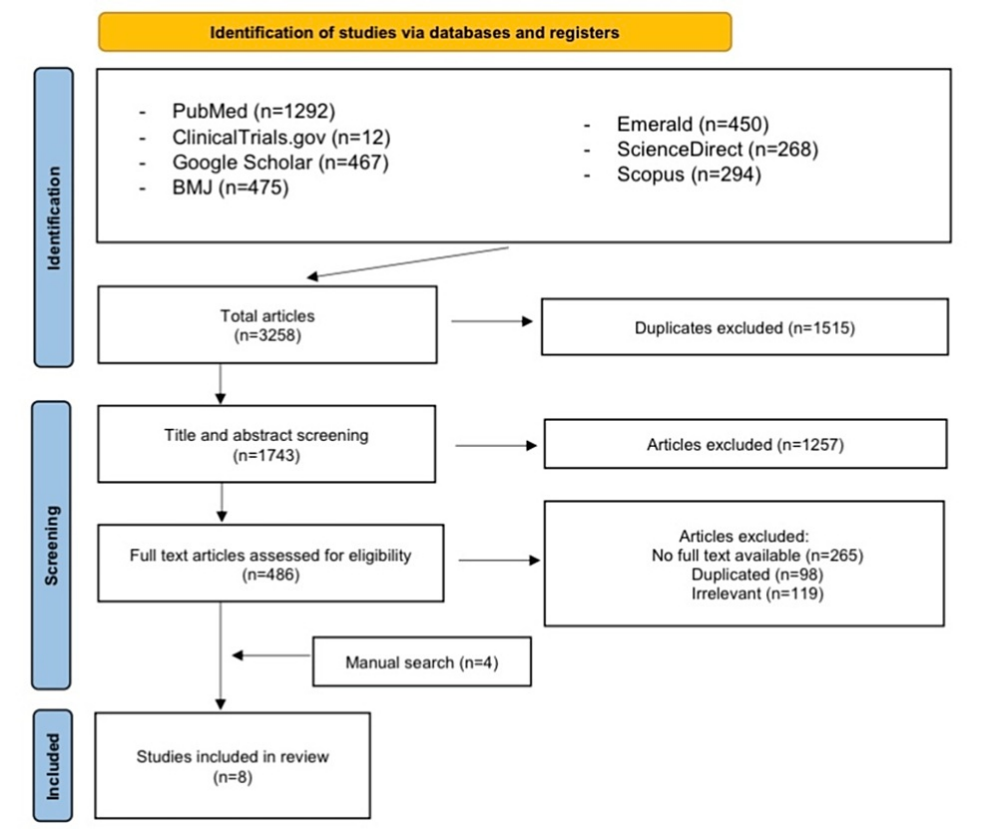
PRISMA flow chart of the systematic review References- [15-22]

The initial search of the electronic databases yielded 3258 potential studies. After exclusions based on titles and abstracts, the full texts of 1743 studies were read for possible inclusion. A total of 8 studies remained for quantitative analysis. Figure 1 summarizes the results of our literature search.

Data Extraction and Quality Assessment of Studies

Two investigators independently searched electronic databases. Studies searched were exported to the EndNote Reference Library software version 20.0.1 (Clarivate Analytics), and duplicates were screened and removed. 

Two investigators independently assessed the quality of the included studies. The risk of biases from RCTs was assessed through Cochrane Collaboration's Tool in seven domains: Adequate Sequence Generation, Allocation Concealment, Blinding of Participants and Personnel, Blinding of Outcome Assessment, Incomplete Outcome Data, Selective Outcome Reporting, Free of Other Bias. Low risk of bias, ambiguous risk of bias, and high risk of bias were the three levels on which the individual domains and overall risk-of-bias judgment were represented. These elements determined whether there was a low, moderate, or high bias risk in the evidence's overall quality. (details in supplement table 1).

**Table 1:** Quality assessment of randomized clinical trials using the Cochrane Collaboration tool References- [15,17,18,20-22]

Study	Random sequence generation	Allocation concealment	Blinding (participants and personnel)	Blinding (outcome assessment)	Incomplete outcome data	Selective reporting	Other sources of bias
Razavi et al., 2018 [15]	Low Risk	Unclear Risk	Unclear Risk	Unclear Risk	Low Risk	Low Risk	Low Risk
Ersoz et al., 2006 [17]	Low Risk	Unclear Risk	High Risk	Unclear Risk	Low Risk	Low Risk	Low Risk
Karoli et al., 2011 [22]	Low Risk	Unclear Risk	High Risk	Unclear Risk	Low Risk	Low Risk	Low Risk
Umpierrez et al., 2004 [18]	Low Risk	Unclear Risk	High Risk	Unclear Risk	Low Risk	Low Risk	Low Risk
Houshyar et al., 2015 [20]	Low Risk	Unclear Risk	Unclear Risk	Unclear Risk	Low Risk	Low Risk	Low Risk
Piters et al., 1977 [21]	Low Risk	Unclear Risk	High Risk	Unclear Risk	Low Risk	Low Risk	Low Risk

The Newcastle-Ottawa Scale (NOS) was used to assess the quality of the cohort studies. NOS scores 1-5 were considered high risk for bias, 6-7 was moderate, and score >7 was considered low risk of bias (details of scoring provided in Table 2).

**Table 2:** Quality assessment of cohorts using the Newcastle-Ottawa Scale References- [16,19]

Study	Selection (Maximum 4)				Comparability (Maximum 2)	Outcome (Maximum 3)			Total score
	Representativeness of the Exposed Cohort	Selection of the Non-Exposed Cohort	Ascertainment of Exposure	Demonstration That Outcome of Interest Was Not Present at Start of Study	Comparability of Cohorts on the Basis of the Design or Analysis	Assessment of Outcome	Was Followed Up Long Enough for Outcomes to Occur	Adequacy of Follow-Up of Cohorts	
Puttha et al., 2010 [16]	1	0	1	1	2	1	1	1	8
Gupta et al., 2018 [19]	1	0	1	1	2	1	1	1	8

Statistical analysis

Review Manager (version 5.4.1; Copenhagen: For all statistical analyses, The Nordic Cochrane Centre, The Cochrane Collaboration, 2020) was used to analyze mild/moderate and severe DKA. A random-effects model was used to combine the data from the various investigations. The results were analyzed by analyzing standard mean difference (SMD) or mean difference (MD) with their respective 95% confidence intervals (CI). The chi-square test was performed to assess any differences between the subgroups. A sensitivity analysis was done to see if any individual study was driving the results and to implore reasons for high heterogeneity. As Higgins et al, the scale for heterogeneity was considered as follows: I2 = 25-50% - moderate; 50-75% - substantial; 75-100% - considerable heterogeneity, and p< 0.1 indicated significant heterogeneity [23]. A p< 0.05 was considered significant for all analyses.

Result

Study Characteristics

Table 3 provides the basic characteristics of the included studies [15-22]. Our analysis included eight published articles. We had 6 Randomized Controlled Trials and 2 Cohort studies. The average age in these studies was 28.9 years. Table 4 shows the baseline biochemical parameters from our patient population.

**Table 3:** Basic characteristics of selected articles N/A*= Not available References- [15-22]

Author	Year	Study type	Sample size	Mean age	Duration of DM (years)	Type of DKA	Intervention	Female%	Net Risk of Bias
Razavi et al. [15]	2018	RCT	25	8.86 ± 0.71	N/A*	6 mild/19 moderate	IV regular insulin (0.05–0.1 unit/kg/hour)	64	Low Risk
Puttha et al. [16]	2010	Observational study	N/A*	Low dose = 8.1 ± 1.63 Standard dose = 10.9 ± 1.48	N/A*	moderate	IV insulin low dose (0.05unit/kg/hour) vs Standard dose (0.1unit/kg/hour)	58	Low Risk
Ersoz et al. [17]	2006	RCT	10	48.8 ± 17.9	4.5 ± 4.3	mid/moderate	IV regular insulin (0.15unit/kg/hour)	60	Low Risk
Karoli et al. [22]	2011	RCT	25	35 ± 11	6.4 ± 5	mild/moderate	IV regular insulin (0.1unit/kg/hour)	36	Low Risk
Umpierrez et al. [18]	2004	RCT	20	39 ± 14	6.9 ± 4	mild/moderate	IV regular insulin (0.1unit/kg/hour)	35	Low Risk
Houshyar et al. [20]	2015	RCT	20	29.25 ± 15.69	N/A*	severe	IV regular insulin (0.1unit/kg/hour)	55	Low Risk
Gupta et al. [19]	2018	Cohort	28	43.3 ± 18.4	N/A*	severe	IV insulin infusion (0.18unit/kg/hour)	48.5	Low Risk
Piters et al. [21]	1977	RCT	26	37 ± 3	N/A*	severe	IV regular insulin Group A = 50 U/hr Group B = 10 U/hr Group C = 2 U/hr	N/A*	Low Risk

**Table 4:** Baseline biochemical factors N/A*= Not available References- [15-22]

Author	Year	BMI (kg/m2)	Plasma glucose (mg/dl)	Arterial pH	Serum bicarbonate (mEq/L)	Urine and serum ketones (positive)	Time to resolution of DKA		Potassium	Factors assessed
Razavi et al. [15]	2018	N/A*	413.88 ± 140.3	<7.3 in 6/ <7.2in 19	<15 in 6/ <10 in 19	N/A*		10.50 ± 5.89 h	N/A*	Plasma glucose, and time of resolution of acidosis.
Puttha et al. [16]	2010	N/A*	Low dose = 26.3 ± 4.3 Standard dose = 26.6 ± 3.7	Low dose = 7.16 ± 0.037 Standard dose = 7.13 ± 0.044	N/A*	N/A*		N/A*	Low dose = 5.1 ± 0.741 Standard dose = 4.9 ± 0.4	Plasma glucose, pH, and potassium levels
Ersoz et al. [17]	2006	N/A*	555.7 ± 42.9	7.18 ± 0.12	10.8 ± 5.7	97.5 ± 50.6		12.7 ± 7.5 h	5.3 ± 0.5	Plasma glucose, pH, potassium levels, and time of resolution of acidosis
Karoli et al. [22]	2011	24 ± 2	679 ± 125	7.18 ± 0.04	13.6 ± 1	N/A*		11 ± 1.6 h	4.8 ± 0.8	Time of resolution of acidosis
Umpierrez et al. [18]	2004	27 ± 9	611 ± 264	7.19 ± 0.08	10.6 ± 4	N/A*		11 ± 4 h	N/A*	Time of resolution of acidosis
Houshyar et al. [20]	2015	22.29 ± 3.42	497.34 ± 102.6	7.09 ± 0.14	6.37 ± 3.49	N/A*		16.91 ± 6.49	4.59 ± 0.59	Time of resolution of acidosis
Gupta et al. [10]	2018	N/A*	480 ± 191	7.1 ± 0.2	8 ± 4	N/A*		12.083 ± 7.81	5 ± 0.9	Time of resolution of acidosis
Piters et al. [21]	1977	N/A*	Group A = 754 ± 62 Group B = 635 ± 84 Group C = 671 ± 95	Group A = 7.14 ± 0.04 Group B = 7.14 ± 0.04 Group C = 7.19 ± 0.04	Group A = 5.8 ± 0.8 Group B = 6.2 ± 0.7 Group C = 7.4 ± 1.0	Group A = 18.5 ± 1.0 Group B = 16.5 ± 1.3 Group C = 16.4 ± 2		N/A*	Group A = 4.9 ± 0.3 Group B = 4.9 ± 0.4 Group C = 5.0 ± 0.3	Plasma glucose, pH, and potassium levels

Publication Bias and Quality Assessment

As there were less than 10 studies, it was not possible to assess the publication bias. All articles have low risk of bias (Table 3).

Results of Meta-Analysis

**Figure 2:**
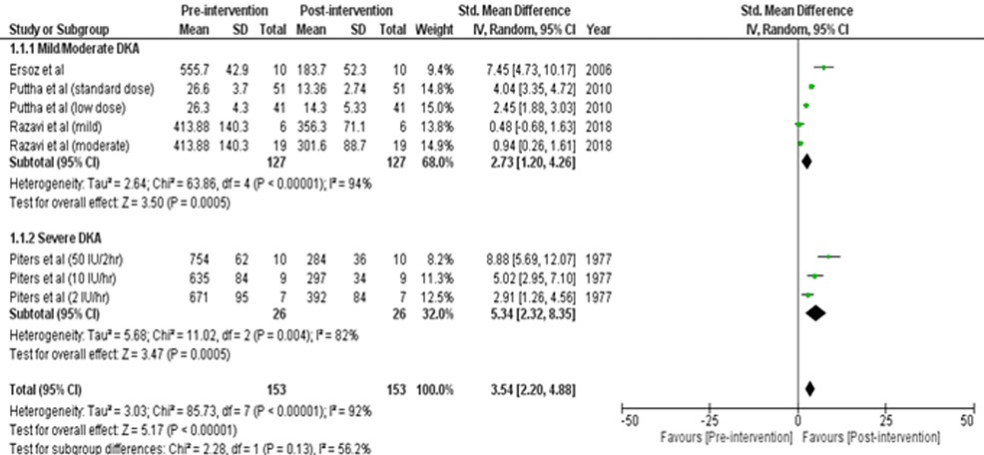
Forest plot showing effect size of regular insulin on plasma glucose

**Figure 3:**
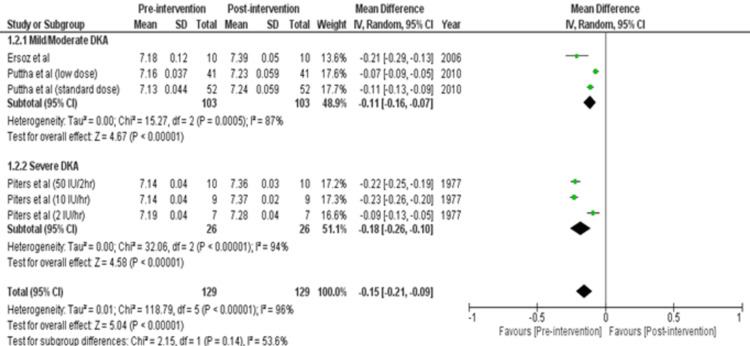
Forest plot showing effect size of regular insulin on pH

**Figure 4:**
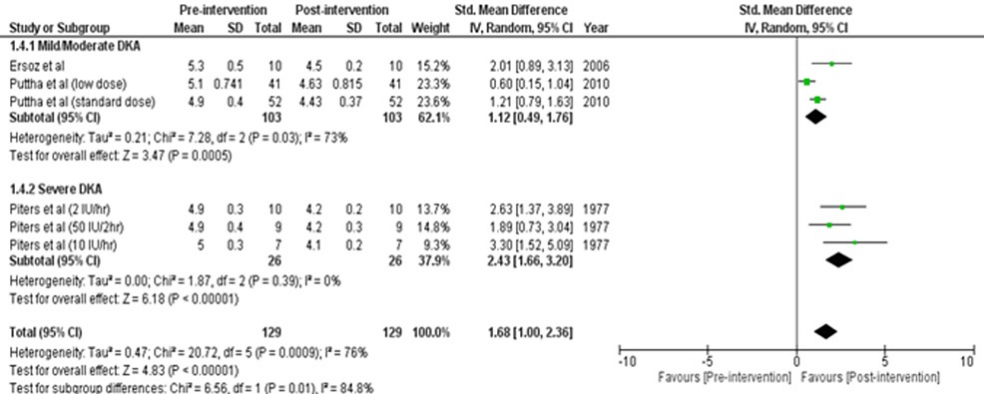
Forest plot showing the effect size of regular insulin on potassium

**Figure 5:**
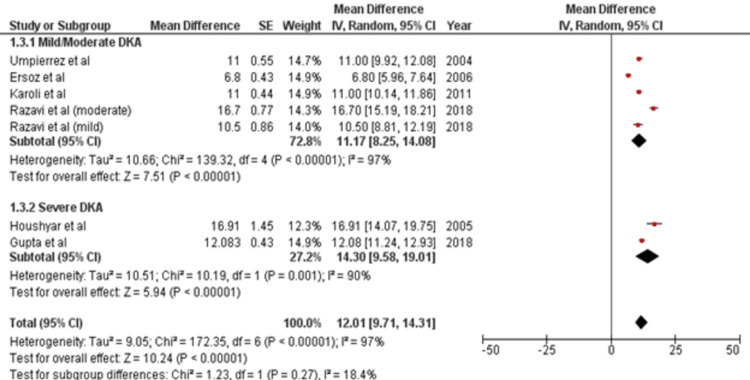
Forest plot showing effect size of regular insulin on time till resolution of acidosis

Review Manager was used for study analysis. Detailed forest plots outlining the effect size of intravenous insulin in Diabetes Ketoacidosis in Plasma glucose (Figure 2), pH (Figure 3), Potassium levels (Figure 4), and time to resolution of acidosis (Figure 5) are provided in the manuscript. 

Plasma Glucose: Out of 8 studies, four studies reported data for plasma glucose [15-17,21]. Pooled results (Figure 2) were based on subgroup analysis by analyzing two intensities of DKA: Mild/Moderate and Severe. There were 127 participants in the mild/moderate group and 26 in the severe group. Analysis showed a statistically significant decrease in Plasma glucose in mild/moderate cases (SMD= 2.73 [1.20, 4.26]; p=0.0005; I2= 94%) and severe cases (SMD= 5.34 [2.32, 8.35]; p=0.0005; I2= 82%). Thus, there was a significant total decrease in plasma sugar (SMD= 3.54 [2.20, 4.88]; p< 0.00001; I2= 92%).

 pH: Out of 8 studies, three studies reported data for plasma glucose [16,17,21]. Pooled results (Figure 3) were based on subgroup analysis by analyzing two intensities of DKA: Mild/Moderate and Severe. There were 103 participants in the mild/moderate group and 26 in the severe group. Analysis showed a statistically significant increase in pH in mild/moderate cases (MD= -0.11 [-0.16, -0.07]; p< 0.00001; I2= 87%) and severe cases (MD= -0.18 [-0.26, -0.10]; p< 0.00001; I2= 94%). Thus, there was a significant total increase in pH (MD= -0.15 [-0.21, -0.09]; p< 0.00001; I2= 96%).

Potassium levels: Out of 8 studies, three studies reported data for potassium levels [16,17,21]. Pooled results (Figure 4) were based on subgroup analysis by analyzing two intensities of DKA: Mild/Moderate and Severe. There were 103 participants in the mild/moderate group and 26 in the severe group. Analysis showed a statistically significant decrease in potassium levels in mild/moderate cases (SMD= 1.12 [0.49, 1.76]; p= 0.0005; I2= 73%) and severe cases (SMD= 2.43 [1.66, 3.20]; p< 0.00001; I2= 0%). Thus, there was a significant total decrease in potassium levels (SMD= 1.68 [1.00, 2.36]; p< 0.00001; I2= 76%).

Time of resolution of acidosis: Out of 8 studies, six reported data for time of resolution of acidosis [15,17-19,20,22]. Pooled results (Figure 5) were based on subgroup analysis by analyzing two intensities of DKA: Mild/Moderate and Severe. Analysis showed that acidosis was resolved more quickly in mild/moderate cases (11.17 hrs [95% CI 8.25, 14.08]) than in severe cases (14.30 hrs [95% CI 9.58, 19.01]). Thus, the total time analyzed for acidosis to resolve was 12.01 hrs [95% CI 9.71, 14.31].

Sensitivity Analysis

By removing one study at a time, a sensitivity analysis was performed to determine the impact of each study on the overall effect. Next, pooled standard mean differences (SMD)/mean differences (MD) were generated for the remaining studies. After any particular study was excluded, no significant change was seen, indicating that the findings were reliable.

Discussion

Summary of Main Findings

Intravenous insulin is one method of managing diabetic ketoacidosis, although there is limited data on its effectiveness and safety in treating diabetic ketoacidosis patients. Intravenous insulin administration is the preferred method of administering insulin to individuals with diabetic ketoacidosis (DKA) [7]. In order to treat DKA, insulin must be administered since it encourages peripheral tissues to utilize glucose, inhibits ketogenesis, and reduces glycogenolysis and gluconeogenesis [24]. Recent recommendations propose starting intravenous insulin as soon as the serum potassium level climbs above (3.3meq/l) and continuing it until the patient is no longer in DKA and may switch to subcutaneous insulin [7]. When the K level is less than 3.3 meq/l, the only time insulin should be stopped replacement with KCL should be performed before insulin is started. The initial insulin dose of 0.1 units/kg should be lowered to 0.05 units/kg if the blood sugar is less than 108 mg/dL. To prevent hypoglycemia, the protocol's order sheet is required to be followed. Every time the blood sugar falls below 72 mg/dL, a bolus of 25 cubic centimeters (cc) of 50% dextrose (D) injection saline can be administered. The alternative is to supplement the current fluids with dextrose 10 % in water (D10W) to raise and keep the blood sugar levels at the desired range. Additionally, insulin therapy aims to reduce plasma glucose by 80 to 100 mg/dL/hr [25]. Numerous studies suggest treating patients with subcutaneous insulin rather than intravenous insulin for uncomplicated, moderate diabetic ketoacidosis may be safer and more cost-effective [18]. The use of intravenous insulin can considerably lower plasma glucose and potassium levels in both mild/moderate instances and severe cases, according to the findings of this review. However, in mild to moderate situations, as opposed to severe cases, it can reduce the period until acidosis resolves more quickly. Patients with DKA should also receive insulin therapy until the condition clears up. DKA resolves when bicarbonate levels are ≥18 mEq/L, and glucose levels are <200 mg/dL [18]. Regarding the severity of cases, no research has previously evaluated the clinical outcomes in DKA patients treated with intravenous insulin. However, in this study, both mild/moderate and severe DKA patients saw a considerable overall increase in pH. Treatment for DKA involves adjusting the IV insulin infusion rate and dextrose concentration (up to 10%, if necessary) to keep blood glucose levels between (150-200 mg/dL)[26]. Despite total body potassium deficiency, mild-to-moderate hyperkalemia is common in hyperglycemic crisis patients. Insulin therapy, acidosis correction, and volume expansion reduce serum potassium concentration [27]. During the treatment of ketoacidosis, hypokalemia and hyperkalemia can be fatal. Because of the risk of acute pre-renal kidney injury associated with severe dehydration, it is recommended that no potassium be prescribed with the initial fluid resuscitation or if the serum potassium level remains above 5.5 mmol/L [28].

Our study revealed a statistically significant decrease in potassium levels in mild, moderate, and severe cases. Thus, there was a significant total decrease in potassium levels. Furthermore, the time of resolution of acidosis was faster in mild and moderate cases than in severe cases.

Limitations

The limitations of the included clinical trials and their methodology should be considered when interpreting this systematic review with meta-analysis (of a retrospective nature and without discarding the possibility of publication bias). Our study was limited by the following factors: (a) there were very few studies and participants in our study; (b) there was considerable heterogeneity in our analysis; (c) we used the number of episodes of DKA in Putha et al. [16] rather than considering the number of children; (d) The type of intravenous insulin was not the same throughout our studies. Nonetheless, these studies were pivotal in obtaining the results of our study, which provides evidence of the advantages of an intravenous insulin intervention in treating DKA. To address the uncertainties around the cost-benefit of this intervention, additional research in the form of multicenter, randomized, double-blind clinical trials with bigger patient populations is necessary. Finally, it would be intriguing to determine whether this strategy applies to all patients with DKA at various stages of severity and, if possible, to formally explore the economic impact that this strategy may have on the national health system by reducing the length of hospital stays and the consumption of resources.

## Conclusions

Based on the findings mentioned, IV insulin is safe and effective in mild/moderate and severe cases of diabetic ketoacidosis (DKA). No significant difference in the efficacy of IV insulin was found between mild/moderate and severe DKA, except in the time of resolution of acidosis, which was faster in mild/moderate DKA than in severe DKA. However, these results should prompt further investigation and attention, as well as further longitudinal research and randomized trials.
